# Does Environmental Regulation Promote Industrial Green Technology Progress? Empirical Evidence from China with a Heterogeneity Analysis

**DOI:** 10.3390/ijerph19010484

**Published:** 2022-01-02

**Authors:** Yanli Ji, Jie Xue, Kaiyang Zhong

**Affiliations:** 1 School of Mathematics and Statistics, Changshu Institute of Technology, Changshu 215500, China; yl012008@cslg.edu.cn; 2 School of Economics, Hangzhou Dianzi University, Hangzhou 310018, China; xjsnow@hdu.edu.cn; 3 School of Economic Information Engineering, Southwestern University of Finance and Economics, Chengdu 611130, China

**Keywords:** environmental regulation, green technology progress, heterogeneity tools, interaction, threshold effect

## Abstract

The complex relationship between environmental regulation and green technology progress has always been a hot topic of research, especially in developing countries, where the impact of environmental regulation is important. Current research is mainly concerned with the impact of the single environmental regulation on technological progress and lacks study on the diversity of environmental regulations. The main purpose of this paper is to examine the heterogeneity of the effects of different types of environmental regulation on industrial green technology progress. As China’s scale of economy and pollution emissions are both large, and the government has also made great efforts in environmental regulation, this paper takes China as the example for analyses. We first use the EBM-GML method to measure the industrial green technology progress of 30 provinces in China from 2000 to 2018, and then apply the panel econometric model and threshold model to empirically investigate the influence of 3 types of environmental regulation. The results show that, first, the impacts of environmental regulation on industrial green technology progress are significantly different; specifically, command-based regulation has no direct significant impact, and autonomous regulation has played a positive role, and market-based regulation’s quadratic curve effect is significant, in which the cost-based and investment-based tool presents an inverted U-sharped and U-sharped, respectively. Second, there may be a weak alternative interaction among different types of environmental regulation. Third, a market-based regulatory tool has a threshold effect; with the upgrading of environmental regulation compliance, the effect of a cost-based tool is characterized by “promotion inhibition”, and that of an investment-based tool is “inhibition promotion”. Finally, the results of regional analysis are basically consistent with those of the national analysis. Based on the study, policy enlightenment is put forward to improve regional industrial green technology progress from the perspective of environmental regulation. This paper can provide a useful analytical framework for studying the relationship between environmental regulation and technological progress in a country, especially in developing countries.

## 1. Introduction

Technological progress, especially green technology-oriented innovation, is an effective means for industrial development to break through the pressure of ecological environment in the long run [1,2,3]. Green innovation has the attribute of public goods, which should be promoted by government environmental regulation [4,5]. The relationship between environmental regulation and green innovation has received more and more attention [6]. Some scholars have proposed that stricter environmental regulations have promoted technological innovation in enterprises [7,8,9]. Some studies have found that environmental regulations have a negative impact on technological progress [10,11]. Some scholars believe that the impact is complex, not just positive or negative [12,13]. In recent years, some scholars have begun to pay attention to the diversity of environmental regulations. In addition, some scholars have paid attention to the impact of the diversity of environmental regulations. Blackman et al. believe that conventional command-and-control environmental regulation often performs poorly in developing countries, and policymakers are trying to explore voluntary regulatory programs [14]. Overall, environmental regulation is being promoted actively [2], and stricter regulatory policies with more diverse tools are imperative in the future [15]. However, there is still relatively little literature on the impact of diversification of environmental regulations on technological progress. Can environmental regulation become an important driving force in industrial green technology? Are there differences among different types of regulation tools? Are there interactions among them? These are very worthwhile questions.

China is the largest developing country. The process of pursuing economic growth is accompanied by a large amount of environmental pollution, so it is a good sample for our research. First, China’s emphasis on environmental regulation began in 1979, a period of initiating reform and opening up. [16]. From the current situation, after more than forty years evolving, environmental problems have become increasingly serious [17]. Evidently, China’s environmental regulations may be not all effective [18]. To explore the above-mentioned issues is of great significance for developing countries such as China, which is entering the stage of high-quality development and facing the dual pressure of economic development and environmental protection. Second, environmental regulation is a comprehensive system, involving legal, economic, social and other issues [19,20,21]. China can provide samples of the diversity of environmental regulations. Third, although there are certain differences in the environmental regulations of different countries, using China as a sample in this study can provide inspiration for other developing countries on the relationship between the diversity of environmental regulation and the progress of green technology.

The rest of the paper is organized as follows: the existing studies are reviewed and research hypotheses are put forward in Section 2; the models, related variables, and data are introduced in Section 3; empirical results and discussion are reported in Section 4; and the conclusion and policy implications are given in Section 5. 

## 2. Literature Review and Research Hypothesis

### 2.1. Literature Review

The analysis of green technological progress begins with the study of green total factor productivity or technological innovation [22]. There are mainly three views on the impact of environmental regulation. On the one hand, the “Restriction Hypothesis” believes that strict environmental regulation leads to a decline of corporate profits, crowding out research and development (R&D) investment and inhibiting technological progress [23,24]. The empirical analysis of the U.S. manufacturing industry [25] and highly polluting industries [26] supports the “crowding out effect” of environmental regulation, which was not conducive to the development of technology. Data from China’s A-share listed companies showed that environmental regulation inhibited enterprise technological innovation [18]. Some scholars also believe that strict environmental regulation would increase enterprise profits, but not enterprise innovation [27].

On the other hand, the “Porter Hypothesis” holds that environmental regulation increases the cost of pollution control in the short term, while it produces an “innovation compensation effect” in the long term to promote technological progress, thereby enhancing the competitiveness of enterprises [28,29]. This view has also been accepted by some scholars. For example, the U.S. petroleum refining industry [30] and Mexican food processing industry [31] used empirical data to confirm that environmental regulation improved the total productivity of the industry. Data from EU economies also confirmed the positive role of market-based environmental regulatory tool on enterprise productivity and innovation [32]. Environmental regulation promoted the technological progress of emission-intensive industries, such as power and chemical industries, and different regulation tools would lock in low or high levels of technology [33]. In China, according to the data grouped by manufacturing industry (high, medium, and low eco-efficiency industries), environmental regulation promoted technology innovation, and there was industry heterogeneity in its effect [34]. Provincial panel data showed that environmental regulation was positively correlated with industrial green productivity [16]. Nationally, environmental regulation played a promoting role in technological progress, and so did the eastern region, but there was a restraining effect in the central and western regions [35]. In addition, micro evidence, such as the analysis of China’s small and medium-sized enterprises, also showed that environmental regulation improved the innovation level [36]. Research on heavy-pollution industries listed companies found that environmental regulation increased the enterprise’s environmentally friendly and non-friendly R&D investment, and improved employee quality, work enthusiasm and innovation, and further improved enterprise productivity [2]. Direct environmental regulation significantly promoted green technology innovation in heavily polluting industries, and technology-capital-intensive industries had a greater effect than labor-intensive industries [37].

Quite a few scholars support that the relationship between environmental regulation and TFP or technological progress is not a simple linear relationship. For example, Shang et al. found that the impact of environmental regulation on provincial green technology innovation was promoted before being hindered [38]. Li et al. argued that the impact of environmental regulation on urban green total factor productivity had a double threshold effect of economic development level, which was reflected in promoting–inhibiting–promoting [39]. Similarly, Du et al. also found that the impact of environmental regulation on urban green technology innovation would change with the level of economic development, but the specific performance was different, which was “inhibition–small influence–significant promotion” [40]. Li et al. investigated the spatial spillover effect of environmental regulation on urban green innovation efficiency based on the spatial model and found that the relationship between environmental regulation and urban green innovation efficiency presents a U-shaped feature [41]. There is also evidence from the industry. For example, Zhou et al. showed that there was a threshold effect based on anti-corruption between the green development level of China’s manufacturing industry and environmental regulation [42]. In addition, a few studies have found that environmental regulation had no significant effect on technological progress or economic growth. For example, an empirical analysis of the more polluting sector of the Canadian manufacturing industry [43], the command regulatory tool in the developed EU economies [32], and China (after entering the new normal) [44] supported this view. 

From the above analysis, the existing results have laid the foundation for this paper, but there are also some shortcomings. Due to differences in research samples, measure variables (such as green innovation and environmental regulation proxy variables) or methods (such as GTFP measurement method), the conclusions differ greatly. When exploring and testing the effects of environmental regulation, only a few failed to take into account the heterogeneity of different types of environmental regulation [16,21]. Most of them started from the perspective of environmental regulation intensity while ignoring the differences of the effect of various regulation tools. In fact, they are different in mechanism and compulsion, thus their policy effectiveness may also be different. In addition, few articles in the literature discuss the interaction of environmental regulatory tools. For these reasons, this paper takes the industrial green technology progress as its research object; divides the environmental regulation into command-based, market-based and autonomous regulation; discusses the influence of three types of environmental regulation and their interaction on the industrial green technology progress; and further considers their threshold effect. This paper attempts to make contributions from the following two aspects. First, we should try our best to accurately measure China’s provincial industrial green technology progress. It is reflected in two aspects. One is data processing, such as the regulation of industrial data adjusted to full-caliber industrial data and considering 23 kinds of energy consumption. The second is the method, including the dynamic depreciation rates and EBM-GML method. Secondly, the panel econometric model and threshold model are used to systematically evaluate the heterogeneity of the effects of three types of environmental regulation on industrial green technology progress in China (the eastern, central and western regions). Among them, the interaction effect of different types of tools and the threshold effect of environmental regulation compliance awareness are some beneficial attempts.

### 2.2. Research Hypothesis

As mentioned above, the impact of environmental regulation on technological progress is summarized as “Restriction Hypothesis” or “Porter Hypothesis”. The Restriction Hypothesis, based on cost compliance, believes that environmental regulation increases enterprise cost, crowds out R&D investment and weakens technological innovation. The Porter Hypothesis, based on technological innovation, believes that environmental regulation forces enterprise innovation, offsets regulatory cost and improves enterprises efficiency. Appropriate environmental regulation not only increases the cost of enterprises, but also stimulates them to carry out innovation activities and promote technological progress. In general, environmental regulation has a smaller impact on high-tech industries with less pollution emissions, and a greater impact on high-pollution industries. Due to the different external environmental constraints in different regions and industries, the difference of environmental regulation intensity will affect the promotion effect of industrial green technology progress.

First, China’s environmental regulation includes three types: command-based, market-based and autonomous regulations. Among them, command-based environmental regulation directly controls enterprises environmental behavior through administrative mandatory orders in the form of laws, regulations and so on. Market-based environmental regulation influences the environmental decision making of enterprises through market means, such as charging. Autonomous environmental regulation mainly improves their own environmental behavior through subjective willingness. There are obvious differences among the three types of regulatory tools in terms of enforcement regulation, compulsion and punishment [16,19,20,21]. Therefore, there may be heterogeneity in improving the effect of industrial green technology progress. By reviewing the literature, it has been found that the conclusions of different environmental regulation measurement variables are different when evaluating the impact on technological innovation [32]. This indicates different regulation tools may have different impact effects. Based on this, we assume that:

**Hypothesis** **1** **(H1).** *There is heterogeneity in the impact of different types of environmental regulation on green technology progress*.

Second, due to the partial overlapping of the regulated objects of different types of environmental regulation, the regulated objects are simultaneously subject to multiple tools. They may have alternative or complementary interaction effects on industrial green technology progress. On the one hand, from the perspective of regulation tools synergy, the coexistence of the three types of tools may have a positive complementary effect on the green technology progress of enterprises. Command-based, market-based and autonomous regulations have their own characteristics, and their effective synergy may bring about complementary effect of “1 + 1 > 2”. On the other hand, from the perspective of the intensity of regulatory instruments, there may be a substitution relationship between the three types of tools. For example, when the mandatory regulation policy is relatively well established and the regulations and rules meet the public expectations, they may reduce the supervision of industrial enterprises’ emission behavior, thereby weakening the intensity of autonomous environmental regulation, resulting in a negative substitution effect on industrial green technology progress. Empirical evidence shows that, when legislation and regulation are absent or ineffective, affected communities are often able to negotiate with neighboring businesses informally to reduce pollution [45]. Based on this, we assume that:

**Hypothesis** **2** **(H2).** *There may be “complementary” or “substitution” effect between different types of environmental regulation*.

Third, the relationship between environmental regulation and green technology progress is not simply linear. When the environmental regulation is at a low level, enterprises are not motivated to carry out innovation activities and occupy R&D investment of energy-saving and emission reduction technology due to the low regulation cost, thus inhibiting the progress of green technology. Increasingly stringent environmental regulations force enterprises to engage in clean technology research and development, thereby promoting green technology progress. Therefore, there may be a U-shaped relationship between environmental regulation and green technology progress. When the level of environmental regulation is low, it plays a negative hindering role. Otherwise, reflecting a positive promotion.

At the same time, when the regulation compliance of regulated objects is different, the impact of environmental regulation on green technology progress may be different, and threshold effect may exist. Some studies have shown that the environmental awareness of relevant personnel has a driving effect on enterprise green technology innovation. For example, Kocabasoglu et al. believed that far-sighted managers would pay more attention to market demands and be willing to reduce the energy consumption level of enterprises to meet customers’ demand for green products [46]. Bansal et al. believed that the environmental awareness of corporate executives would help them realize the seriousness and urgency of environmental problems, and then prompt enterprises to take active measures [47]. Zhang et al. found in their research on Chinese enterprises that senior executives’ environmental awareness was a key factor affecting enterprises’ green behavior [48]. In addition, Duarte’s study found that corporate executives would form an environmental awareness due to their community’s concern on environmental issues, thus influencing corporate environmental behavior [49]. There is also environmental pressure from enterprise suppliers. Zhang et al. found that environmental pressure from enterprise suppliers could significantly promote enterprises’ environmental management practices [50]. The environmental awareness of corporate executives, community members and suppliers can be summarized as the awareness of environmental regulation compliance. Based on this, we assume that:

**Hypothesis** **3** **(H3).** *There may be a non-linear relationship between environmental regulation and industrial green technology progress, and a threshold effect of environmental regulation compliance*.

**Figure 1:**
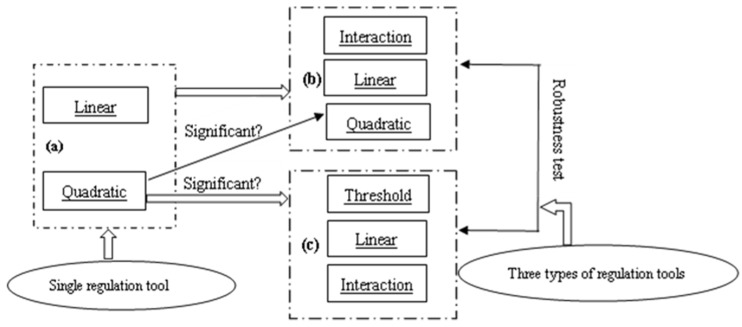
Research framework: (**a**) Do linear and quadratic effects of a single regulatory tool hold true?; (**b**) Whether interaction effects of different regulatory tools are significant (in the framework of linear and quadratic effects, only containing regulatory tools that pass the quadratic significance test)?; (**c**) Whether threshold effects of regulatory tools with quadratic effect are significant (in the framework of linear and interaction effects)?

Based on the above assumptions, the research framework of this paper is constructed, as shown in Figure 1.

## 3. Model and Data

### 3.1. Empirical Model

This paper considers whether environmental regulation has an impact on green technology progress: Is it a non-linear relationship? If so, the threshold effect is further analyzed. At the same time, is there any interaction effect between different types of environmental regulation? Therefore, three types of models are constructed.

#### 3.1.1. Benchmark Model (i.e., Situation (a) of Research Framework)

The linear and quadratic regression models of environmental regulation on industrial green technology progress are used to determine whether the “specific mode of action” between them is significant. The model is as follows:

(1) $$Gy_{it}=\alpha_{0a}+\beta_{0a}Er_{ait}+\gamma_{0a}Er_{ait}^{2}+\theta_{0a}CX_{it}+\varepsilon_{ait}$$

In Equation (1), *i* and *t* represent the province and year, respectively; a represents the type of environmental regulation. $Gy_{it}$ denotes industrial green technology progress; $Er_{ait}$ measures the intensity of regulation tool; $CX_{it}$ is a set of control variables. $\alpha_{0a},\beta_{0a},\gamma_{0a}$ and $\theta_{0a}$ are the expected coefficients, and $\theta_{0a}$ is a vector; $\varepsilon_{ait}$ is the random disturbance. We tested $Er_{ait}$ one by one. If $\beta_{0a}\neq 0$ and $\gamma_{0a}=0$, it shows that $Er_{ait}$ has a significant linear effect on $Gy_{it}$. Similarly, if $\gamma_{0a}\neq 0$, it shows the “parabolic” relationship between $Er_{ait}$ and $Gy_{it}$ holds.

#### 3.1.2. Interaction Effect Model (i.e., Situation (b) of Research Framework)

Three types of regulatory tools and the intersection terms of them are placed in the same model to investigate their interaction. The model only includes square terms that pass the significance test. The model is as follows:

(2) $$Gy_{it}=\alpha_{1a}+\sum_{a=1}^{q}\beta_{1a}Er_{ait}+\sum_{a=1}^{q}\gamma_{1a}Er_{ait}^{2}+\sum_{d=1}^{q}\sum_{w<d}\eta_{1dw}Er_{dit}*Er_{wit}+\theta_{1a}CX_{it}+\upsilon_{ait}$$

In Equation (2), except for the same variables as (1), q is the number of types of regulation tools. $Er_{dit}$ and $Er_{wit}$ represent the intensity of regulation tool *d* and *w*. $\alpha_{1a},\beta_{1a},\gamma_{1a},\eta_{1dw}$ and $\theta_{1a}$ are the expected coefficients, and $\theta_{1a}$ is a vector; $\upsilon_{ait}$ is the random disturbance. There is an interaction effect between $Er_{d}$ and $Er_{w}$ if $\eta_{1dw}\neq 0$. Furthermore, it is a complementary effect if the coefficient is greater than 0; otherwise, it is a substitution effect. 

#### 3.1.3. Threshold Effect Model (i.e., Situation (c) of Research Framework)

If there is a quadratic mode of regulation tools, it is necessary to further test whether there is a threshold effect of regulatory compliance. Drawing on the ideas of Hansen [51], the panel threshold regression model is constructed:

(3) $$\begin{array}{c} Gy_{it}=\alpha_{2a}+\beta_{2a1}Er_{ait}(Ze_{it}\leq \tau_{{}_{1}})+\beta_{2a2}Er_{ait}I(\tau_{1}<Ze_{it}\leq \tau_{2})+\cdots +\beta_{2ak}Er_{ait}I(\tau_{k-1}<Ze_{it}\leq \tau_{k})\\ +\beta_{2ak+1}Er_{it}I(Ze_{it}>\tau_{k})+\sum_{d=1}^{q}\sum_{w<d}\eta_{2dw}Er_{dit}*Er_{wit}+\theta_{2a}CX_{it}+\mu_{ait} \end{array}$$

In Equation (3), except for the same variables as in Equation (2), $Ze_{it}$ is the threshold variable, indicating the degree of compliance with environmental regulations. I(⋅) is an indicator function; $\tau_{1},\tau_{2},\cdots \tau_{k}$ are threshold values. The influence coefficient has changed if $Ze_{it}$ is greater than $\tau_{j}$. $\alpha_{2a},\beta_{2a1},\beta_{2a2}，\cdots \beta_{2ak+1},\eta_{2dw}$ and $\theta_{2a}$ are the expected coefficients, and $\theta_{2a}$ is a vector; $\mu_{ait}$ is the random disturbance.

### 3.2. Variables and Data

#### 3.2.1. Explained Variable

The measurement of industrial green technological progress is one of the core links of this paper. In terms of the calculation method, the existing literature adopts two ideas. One is to select a single indicator, such as per capita income [52], the number of green patent grants [37,38] and the sum of green patent grants and green technology awards as proxy variable [42]. The other is to use green total factor productivity (GTFP) or its decomposition term, such as the green total factor productivity and the global Malmquist–Luenberger (GML) productivity index [2,40,53], or their decomposition of technological progress [41,44,54]. The latter minimizes measurement errors. Compared with single proxy variable, GTFP or its decomposition term measurement method is more comprehensive. Therefore, this paper is used it to measure industrial green technology progress. Due to its combination with radial and non-radial model, the epsilon-based measure (EBM) model has a more practical applicability [55,56]. So, EBM model is selected to measure technical efficiency. The details are as follows:

(4) $$\begin{array}{l} \psi =\min\frac{\xi -\omega_{x}\sum_{u=1}^{m}\frac{\varpi_{u}^{-}s_{u}^{-}}{x_{uh}}}{\varphi +\omega_{y_{G}}\sum_{j=1}^{n}\frac{\varpi_{j}^{+}s_{j}^{+}}{y_{Gjh}}+\omega_{y_{B}}\sum_{z=1}^{l}\frac{\varpi_{z}^{-}s_{z}^{-}}{y_{Bzh}}}\\ s.t.\left\{ \begin{array}{l} X\delta +s_{u}^{-}=\xi x_{h},\begin{array}{cc} & u=1,2,\cdots ,m \end{array}\\ Y_{G}\delta -s_{j}^{+}=\varphi y_{Gh},\begin{array}{cc} & j=1,2,\cdots ,n \end{array}\\ Y_{B}\delta +s_{z}^{+}=\varphi y_{Bh},\begin{array}{cc} & z=1,2,\cdots ,l \end{array}\\ \delta \geq 0,s_{u}^{-},s_{j}^{+},s_{z}^{-}\geq 0 \end{array}\right. \end{array}$$

In Equation (4), $X,Y_{G}$ and $Y_{B}$ represent *m* kinds of inputs, *n* kinds of expected outputs and *l* kinds of non-expected outputs. *H* is the number of decision units. ψ(0≤ψ≤1) is the optimal efficiency value; $\varpi_{u}^{-},\varpi_{j}^{+},\varpi_{z}^{-}$ and $s_{u}^{-},s_{j}^{+},s_{z}^{-}$ are the weights and slacks of the *Uth* input, the *Jth* expected output and the *Zth* undesirable output, respectively. ω(0≤ω≤1) synthesizes the radial efficiency value and ξ are important parameters of non-radial slack variables.

Further, using the practice of Zhou et al. [57] for reference, this paper has constructed GML index based on technical efficiency and decomposed it. The details are as follows:

(5) $$\begin{array}{l} G^{t,t+1}(x^{t},y_{{}^{G}}^{t},y_{B}^{t};x^{t+1},y_{G}^{t+1},y_{B}^{t+1})=\frac{\psi^{G,t+1}（x^{t+1},y_{G}^{t+1},y_{B}^{t+1}）}{\psi^{G,t}（x^{t},y_{{}^{G}}^{t},y_{B}^{t}）}\\ =\frac{\psi^{t+1}（x^{t+1},y_{G}^{t+1},y_{B}^{t+1}）}{\psi^{t}（x^{t},y_{{}^{G}}^{t},y_{B}^{t}）}\times \frac{\psi^{G,t+1}（x^{t+1},y_{G}^{t+1},y_{B}^{t+1}）/\psi^{t+1}（x^{t+1},y_{G}^{t+1},y_{B}^{t+1}）}{\psi^{G,t}（x^{t},y_{{}^{G}}^{t},y_{B}^{t}）/\psi^{t}（x^{t},y_{{}^{G}}^{t},y_{B}^{t}）}\\ =Gec^{t,t+1}\times Gtc^{t,t+1} \end{array}$$

In Equation (5), $\psi^{G，t}$ and $\psi^{G，t+1}$ represent the global efficiency value of *t* period and *t* + 1 period, respectively. $G^{t,t+1}$ represents the green total factor productivity index, and it is decomposed into green technology efficiency index $\left(Gec^{t,t+1}\right)$ and green technology progress index $\left(Gtc^{t,t+1}\right)$. Between them, green technology progress measures the real level of technology. At the same time, taking the year 2000 as 1, then multiplying the green technology progress index cumulatively to obtain the industrial green technology progress (Gy).

According to the EBM-GML method, this paper has constructed an index system including capital, labor and energy input, industrial desirable and undesirable output. Among them, capital and labor force are measured by the industrial fixed capital stock and the number of industrial employees. Energy is measured by industrial terminal energy consumption. Desirable industrial output is measured by the total industrial output value, while non-desirable outputs are measured by the emissions of three industrial wastes (wastewater, waste gas and solid waste). See Table 1 for details.

Furthermore, input–output indicator data (30 Provinces in China from 2000 to 2018, see Section 3.2.4 for details) are described. The original data of fixed capital stock, number of employees, and gross output value are calculated as industrial enterprises above the designated size, which need to be adjusted to all industrial enterprises (hereinafter, “industrial enterprises above the designated size” is referred to as “enterprises above the designated size” and “all industrial enterprises” as “industry-wide enterprises”). The key to adjusting them is to estimate the proportional coefficient. Considering the scope of enterprises above the designated size changed in 2007 and 2011, the estimation is divided into three periods, 2000–2006, 2007–2010 and 2011–2018. The specific steps are as follows. First, the number of industry-wide employees is divided by the number of employees in enterprises above the designated size in the corresponding years, and the coefficients of 2004 and 2008 are calculated (since the first and second national economic censuses were conducted in 2004 and 2008 and provided the number of industry-wide employees in each province, the two years were selected as the basic for coefficient adjustment). Then, assuming the coefficients change linearly during the study period, the adjustment coefficients from 2000 to 2006 are further estimated based on the coefficient 2004, and the coefficients from 2007 to 2010 based on the coefficient 2008. Thirdly, the number of industry-wide employees in 2011 is calculated based on its growth rate in 2010 and divided by the number of employees in the industrial enterprises above the designated size to obtain the annual adjustment coefficient. Similarly, the proportional coefficients are estimated from 2012 to 2018. Finally, the number of employees in the industrial enterprises above the designated size is multiplied by the adjustment coefficient, that is the number of industry-wide employees $X_{2it}$. In the same way, the gross output value of the enterprises above the designated size is unified into the gross output value of industry-wide enterprises and divided by the ex-factory price index of industrial products of each province, which is the gross industrial output value $Y_{Git}$.

The fixed capital stock is estimated by the perpetual inventory method, i.e., $X_{1it}=I_{it}/IP_{it}+(1-\kappa_{it})X_{1it-1}$, where $X_{1it},I_{it},$ $IP_{it}$ and $\kappa_{it}$ represent the fixed capital stock, total fixed capital formation, fixed asset investment price index and capital depreciation rate of province *i* in year *t*, respectively. According to the proportion coefficient estimation method above, the original price of fixed assets is adjusted into industry-wide enterprises and making the difference between the data of the current period and the data of previous period to obtain $I_{it}$. For $\kappa_{it}$, it is assumed that the industry-wide enterprises are equal to the enterprises above the designated size. Therefore, this paper has used the depreciation and the original price of fixed assets of the enterprises above the designated size for its calculations. For the initial capital stock $X_{1i2000}$, the net value of fixed assets in 1999 can be unified into the industry-wide caliber by the adjustment coefficient.

For the industrial terminal energy consumption $X_{3it}$, by means of the conversion coefficients of different kinds of energy standard coal, the industrial terminal energy physical consumptions of 23 kinds of energy in each province in each year are converted into the standard quantity and summed up. The data of industrial wastewater emissions $Y_{B1it}$, SO_2_ emissions $Y_{B2it}$ and fixed waste emissions $Y_{B3it}$ are directly gathered. 

#### 3.2.2. Main Explanatory Variables

Environmental regulation is the core explanatory variable in this paper. Based on the relevant literature, two variables are selected to measure from the three types of environmental regulation, namely, command-based, market-based and autonomous type, so as to show the reliability of the conclusion. See Table 2 for details.

Two points need to be noted. Firstly, there are many market-based environmental regulation indicators, such as pollution emission intensity, the proportion of total pollution control investment in value added, and fiscal expenditure on environmental protection. Based on investment and cost regulation tools, the intensity of pollution control investment completion and per capita pollution charges are chosen. Secondly, the number of laws and regulations issued by local governments and the number of proposals made by the National People’s Congress (NPC) and the Chinese People’s Political Consultative Conference (CPPCC) are not related to the scale of population or output value, so there is no need for “relativization”.

In addition, to measure the threshold effect of environmental regulation, it is necessary to set the threshold variable of environmental regulation compliance degree $Ze_{it}$. Since this paper considers citizens’ awareness of compliance with environmental regulations, using the practice of Zhong et al. (2019) [58] for reference, the reciprocal of urban per capita domestic sewage emission is adopted. The higher the value is, the higher the compliance consciousness of environmental regulation. 

#### 3.2.3. Control Variables

This paper selected seven control variables. They are: (a) R&D intensity $\left(Kc_{it}\right)$ is the ratio of internal R&D expenses of the enterprises above the designated size to its added value; (b) level of opening up $\left(Tr_{it}\right)$ is the ratio of total import and export to GDP; (c) foreign direct investment $\left(FDI_{it}\right)$ is measured by the ratio of industrial foreign direct investment to GDP, and the total import and export and foreign direct investment are converted into the RMB value by the exchange rate of USD to RMB, then divided with the gross regional product; (d) governance transformation $\left(Zl_{it}\right)$ is measured by the ratio of the main business income of industrial private enterprises (including private, foreign investment and Hong Kong, Macao and Taiwan investment industrial enterprises) to state-owned and state-controlled enterprises above the designated size; (e) industrial structure $\left(S_{it}\right)$ is the proportion of value added of the tertiary industry; (f) energy price $\left(P_{it}\right)$ is measured by fuel and power purchasing price index, taking 2000 as the base period, the fuel and power purchasing price indexes in the remaining years are cumulatively multiplied; (g) energy consumption structure $\left(Co_{it}\right)$ is the proportion of coal in the total terminal energy consumption. 

#### 3.2.4. Data Descriptive Statistics

The above relevant data came from China Statistical Yearbook, China Industrial Economic Statistical Yearbook, China Environmental Yearbook, China Population and Employment Statistics Yearbook, China Statistical Yearbook on Science and Technology, China Energy Statistical Yearbook, and Provinces’ Statistical Yearbooks from 2001 to 2019. 

In view of the availability of data, this paper studies the impact of different types of environmental regulation on the progress of industrial green technology in 30 provinces (excluding Tibet, Hong Kong, Macao and Taiwan) in China from 2000 to 2018. From the perspective of geographical division, 30 provinces belong to the eastern (Beijing, Tianjin, Hebei, Liaoning, Shanghai, Jiangsu, Zhejiang, Fujian, Shandong, Guangdong and Hainan), central (Shanxi, Jilin, Heilongjiang, Anhui, Jiangxi, Henan, Hubei and Hunan) and western regions (Inner Mongolia, Guangxi, Chongqing, Sichuan, Guizhou, Yunnan, Shaanxi, Gansu, Qinghai, Ningxia and Xinjiang).

China’s three regions have obvious characteristics. Overall, the eastern economy is more developed, followed by the central and the western underdeveloped. It is the same in industrial structure, the level of opening to the outside world, etc. As shown in Table 3, the average values of the proportion of tertiary industry (S), R&D intensity (Kc), opening-up level (Tr), foreign direct investment (FDI) and governance transformation (Zl) decrease successively in the eastern, central and western regions, and the eastern region is higher than the national average, while the central and western regions are lower than the average. The gap between the three regions is obvious, especially the level of opening to the outside world and foreign direct investment. Due to different resource endowments, there is also a difference in the energy consumption structure. The proportion of coal consumption in the central region is relatively high, at 9.61 percentage points higher than the national average, while in the eastern region, the proportion of coal consumption is 9.02 percentage points lower than the national average. From a regional perspective, except for the export-oriented economy, the differences in the eastern provinces are higher, which can be seen from the variation coefficient of variables.

However, the regional performance of the industrial green technology level is different, with the eastern, western and central regions gradually increasing. The average value of industrial green technological progress (Gy) is 1.04, 1.08 and 1.11, respectively. The differences between central provinces and western provinces are greater, while the differences of eastern provinces are relatively small. Because the coefficients of variation of Gy are higher in the central and western regions. In terms of environmental regulation, the performance of different types of regulation varies among regions. For example, the per capita, the pollution charge (*Er*_3_) is higher in the eastern region, and similar in central and western regions. The intensity of pollution control investment completion (*Er*_4_) is higher in the western region, while the central and eastern regions are closer. These show that the intensity of environmental regulation in the three regions varies with different regulatory tools. Furthermore, the differences of environmental regulation intensity within the region are relatively large in the central and western regions, which can be seen from the variation coefficient of variables, except for *Er*_6_. However, the mean levels of compliance degree of environmental regulation are basically the same in the three regions.

## 4. Results and Discussion

In this section, the results are interpreted. We have analyzed the linear and quadratic effects of regulatory tools, the interaction effects of different types of regulatory tools, the threshold effects of regulatory tools with quadratic effect, and the robustness test from a regional perspective. 

### 4.1. Baseline Regression

First, we estimate Equation (1). Panel data models contain pooled OLS, fixed-effect, and random effect models. Generally, the likelihood (LR) test and Hausman test are used to determine the form of the models. LR test is selected to identify of pooled OLS or fixed-effect model, and Hausman test for the identification of fixed-effect or random effect model. In this paper, the above tests are to complete the selection of panel model in turn and the results are listed (rows (8)–(9) of Table 4, Table 5 and Table 6). Based on the results in Table 4, Table 5 and Table 6, LR tests reject the null hypothesis at the significant level of 1% for all models. Therefore, it is reasonable for these models to choose the fixed-effect model. For Hausman tests, several models, namely models T11, T12, T21, T22, T41, T42, T51, T52, T61 and T62, reject the null hypothesis at the significant level of 1% or 5%, indicating that fixed-effect models are suitable for them. In comparison, models T31 and T32 are not able to reject the null hypotheses of random effect, suggesting that they are based on random effects. Regression equations are sorted out and the primary and square terms coefficients of regulatory tools are listed in Table 4, Table 5 and Table 6 (all concepts and the coefficients of control variables are omitted to save space).

As can be seen from Table 4, command-based environmental regulation has no significant effect on industrial green technology progress. After introducing the control variables CX, $Er_{1}$ does not pass the significance test. Therefore, the linear relationship between $Er_{1}$ and Gy is not established. After adding $Er_{1}^{2}$, the coefficients of $Er_{1}$ and $Er_{1}^{2}$ are not significant. It shows the quadratic relationship between them is not established either. For $Er_{2}$, the conclusion is the same. Therefore, command-based regulation has no direct impact on industrial green technology progress. This result is the same as the conclusion of Santis et al. [32] and is not inconsistent with the views of Swaney [59] and Fisher et al. [60]. They agreed that command-based regulatory tools could not effectively stimulate innovation and thus did not promote technological progress. For a long time in the past, China’s environmental laws, regulations and other command-based tools did not clearly define the regulatory supervision authority, and the implementation of the local government environmental regulation binding force was low [38]. Moreover, the policy was too rigid, and enterprises had little space for independent choice. Once the regulatory standards were reached, there would be no R&D investment motivation [16]. As a result, such regulation has no significant effect on enterprise green technology progress.

Table 5 shows that market-based environmental regulation has a significant impact on the progress of industrial green technology: inhibition or promotion. The coefficient of $Er_{3}^{2}$ is significantly negative, indicating that an inverted U-shaped relationship is between $Er_{3}$ and Gy. However, the relationship between $Er_{4}$ and Gy is U-shaped, and the coefficient of $Er_{4}^{2}$ is significantly positive. Therefore, it can be seen that market-based regulation has a significant quadratic effect on industrial green technology progress, but the form is different. Cost-based tool $Er_{3}$ rises first and then suppresses, which is consistent with the conclusions of Zhang et al. [21] and Adam et al. [61], and unanimously agreed with the first rise and then suppress effect of sewage charges. In fact, many scholars support that market-based regulatory tools have more evident effects than command-based tools. However, the investment-based tool $Er_{4}$ suppresses first and then rises, which is contrary to the research conclusion of Shang et al. [38]. The reason may be their different research objects. Shang et al. discussed China’s provincial green innovation (the number of green patents granted), while this paper studies China’s provincial industrial green technology progress. Both are closely related, but there are also differences. The result is similar to Zhou et al.’s conclusion that investment-type tools could promote the green development of manufacturing [42]. 

The significant effect of market-based regulation may be due to the fact that such market-based regulation tools allow enterprises to choose the optimal development strategy independently under the premise of environmental regulation. In terms of cost-based tool, R&D resources of enterprises cannot be squeezed under low pollution charges; and within a certain range, increasing the cost gradually will stimulate enterprises to increase R&D investment and improve their green technology innovation. On the contrary, R&D resources of enterprises are squeezed under high pollution charges, thus hindering the green innovation of enterprises. Therefore, the effect of the cost-based tool shows an inverted U-shaped characteristic of “raises first then falls”. For the investment-based tool, due to the lack of pollution control equipment and technology in the early stage, the investment of enterprises’ pollution control funds will squeeze their R&D investment, which is not conducive to improving enterprises green technology. With the accumulation of pollution control facilities and clean technologies, the income effect of regulation has increased enterprises profits. Under the tighter environmental policies, enterprises will further increase investment in innovation, and then promote the progress of green technology. As a result, the effect of investment tools on industrial green technology progress shows a U-shaped characteristic of “first restrain then accelerate”. 

Table 6 displays that autonomous environmental regulation has promoted industrial green technology progress. $Er_{5}$ and $Er_{6}$ both pass the significance test, and the coefficients are greater than 0. After introducing $Er_{5}^{2}$ and $Er_{6}^{2}$ successively, none of the coefficients pass the test. It shows that the positive linear relationship for $Er_{5}$ and $Er_{6}$ is true, while the quadratic curve relationship is not significant. This conclusion is not completely consistent with that of Zhang et al. [21]. The latter not only supported the positive of autonomous tools on urban green innovation, but also agreed with its inverted U-sharped effect. They chose different variables of regulatory tools. A possible explanation for the increase in intensity of $Er_{5}$ and $Er_{6}$ having promoted the progress of industrial green technology is that the increase in residents’ awareness of environmental protection and preference for green products has forced enterprises to increase investment in clean technology innovation, thus promoting green technology progress of enterprises. 

In summary, the effect of China’s environmental regulation on industrial green technology progress changes with the types of regulation. Command-based regulation has no direct significant effect and autonomous regulation positively promotes industrial green technology progress, while market-based regulation has a significant quadratic effect, and the specific forms of cost and investment tools are different. Thus, Hypothesis 1 is verified.

### 4.2. Interaction and Threshold Effect Regression

#### 4.2.1. Interaction Regression

Next, we introduce the interaction term and estimated Equation (2). The six regulation tools are divided into two groups according to their types: $Er_{1},Er_{3},Er_{5}$ and $Er_{2},Er_{4},Er_{6}$. Since the quadratic effects of command-based and autonomous environmental regulations on industrial green technology progress are not significant, the only square terms of market-based tools are included. According to the previous practice [62], the selection of pooled OLS, fixed-effect or random effect models is completed. For model T1, the LR test significantly rejects the null hypothesis, indicating that the fixed-effect model is suitable, and the Hausman test also significantly rejects the null hypothesis, which suggests the fixed-effect model is reasonable for it. Therefore, model T1 selects the fixed-effect model. Similarly, for model T2, the LR test significantly rejects the null hypothesis, while the Hausman test is not able to reject the null hypothesis, so the random-effect model is selected. Table 7 lists the specific regression results.

Table 7 shows that, for models T1 and T2, only $Er_{3}*Er_{5}$ and $Er_{2}*Er_{6}$ pass the significance test in the intersection terms of the six regulation tools, and the coefficients are −0.0003 and −0.0001, respectively. It shows that the inhibitory effects of them are significant, but relatively weak. Therefore, there may be a negative substitution effect between autonomous and market-based environmental regulations, and autonomous and command-based environmental regulations, while the interaction between command-based and market-based environmental regulations does not exist. Thus, in the evolution of industrial green technology, there is not a positive complementary effect, but a weak substitution effect between different types of environmental regulation. Therefore, Hypothesis 2 is confirmed.

#### 4.2.2. Threshold Regression

As can be seen from the above analysis, market-based regulation has a significant non-linear impact on industrial green technology progress. Therefore, the threshold effect of environmental regulation was further tested from the perspective of compliance degree.

We estimate Equation (3). Using the bootstrap method, the single, double and triple threshold effects of market-based regulation tools are tested. The results are listed in Table 8.

As shown in Table 8, single threshold models are both significant, while double threshold and triple threshold models are not significant. Therefore, $Er_{3}$ and $Er_{4}$ are analyzed based on single threshold models. Meanwhile, single threshold estimators are within the corresponding 95% confidence interval, indicating that the threshold estimates are authentic [51]. The estimated threshold regression models are further shown in Table 9, in which model T3 presents the regression results of *Er*_3_, and model T4 for those of *Er*_4_.

Table 9′s model T3 shows that there is a threshold effect of market-based environmental regulations on industrial green technology progress due to the environmental regulation compliance degree (Ze). When Ze≤0.034, $Er_{3}$ has a significant positive impact on Gy and the coefficient is 0.0019. The coefficient decreases to −0.0136 when Ze>0.034, which is consistent with the research conclusion of Xie et al. [16]. It can be concluded that, when the compliance awareness of environmental regulations is low, cost-based tool positively promotes the progress of industrial green technology and, when increases to a certain level, it hinders the progress of industrial green technology, and the hindering effect becomes stronger. Furthermore, the current cost-based tool in 30 provinces is hampering industrial green technology progress.

On the other hand, model T4 of Table 9 indicates $Er_{4}$ has a significantly negative effect on Gy with the coefficient of −0.1494 when Ze≤0.014. The coefficient increases to 0.0282 and is not significant when Ze>0.014. This shows that, when the regulatory compliance degree is switched to a specific level, the significant hindering effect of investment-based tool to industrial green technology progress disappears. At present, except for Beijing, Liaoning, Shanghai, Jiangsu and Zhejiang, the promoting effect of investment-based tool in the other 25 provinces is not evident. Therefore, hypothesis 3 is verified.

The comparison shows that the conclusions of the threshold and interaction regressions are consistent. For example, the threshold effect of $Er_{3}$ shows the characteristics of raises first then falls, which is consistent with the inverted U-shaped between $Er_{3}$ and Gy; the threshold effect of $Er_{4}$ shows the characteristics of first restrain then accelerate, which is consistent with the U-shaped between $Er_{4}$ and Gy. The coefficients of autonomous regulation tools $(Er_{5}$ and $Er_{6})$ are significantly positive, while the coefficients of imperative regulation tools $(Er_{1}$ and $Er_{2})$ do not pass the significance test. In the cross terms, only the coefficient of *Er*_3_∗*Er*_5_ is significantly less than 0. The conclusions of both regressions are the same.

For control variables, except for Kc, the other six variables pass the significance test. The coefficients of Tr, S and are negative, while the coefficients of FDI,Zl and P are positive. This is true for models T1, T2, T3, and T4. Therefore, in the evolution of regional industrial green technology, the level of opening up, the proportion of tertiary industry and the proportion of coal consumption have inhibiting effects, while foreign direct investment, governance transformation and energy prices play a significant positive role. In particular, the impacts of governance transformation and energy prices are relatively high, which are the embodiment of “efficient markets”. Environmental regulation is the performance of “promising government”. Therefore, the combination of an efficient market and a promising government is very important to industrial green technology progress.

### 4.3. Further Analysis: Regional Comparison

Considering the regional differences of industrial green technology level and environmental regulation intensity, the regional diversity of the relationship between them is analyzed. Since the conclusion of a single regulation tool analysis is quite consistent with that of interaction effect regression, the regional discussion only focuses on interaction effect model and threshold effect model, dividing 30 provinces into the eastern, central and western region. The regional division is described in Section 3.2.4.

#### 4.3.1. Interactive Regression

The selection and estimation results of panel regression models of interaction effects in different environmental regulation tools in the three regions are summarized in Table 10. Models E1 and E2 are for the eastern region, models C1 and C2 for the central region, and models W1 and W2 for the western region.

Table 10 shows that model W2 is reasonable for the random effect model, and the other five panel models are all suitable for fixed-effect models. For specific identification basis, please refer to Section 4.1 or Section 4.2.1.

Models E1 and E2 show that the coefficients of $Er_{1}$ and $Er_{2}$ do not pass the significance test, and the coefficients of $Er_{5}$ and $Er_{6}$ are significantly positive. It can be concluded that the autonomous regulation promotes industrial green technology progress in the eastern region, while the command-based regulation has no significant effect. The coefficient $Er_{3}$ is significantly positive, and $Er_{3}^{2},Er_{4},Er_{4}^{2}$ do not pass the significance test, indicating that there is no “quadratic curve” effect between market-based environmental regulation and industrial green technology progress and the linear relationship is uncertain. At the same time, only the coefficient of $Er_{3}*Er_{5}$ is significantly negative among the six cross terms, suggesting that there may be “substitution” interaction between market-based and autonomous regulatory tools in the eastern region.

Models M1 and M2 show that $Er_{4},Er_{4}^{2},Er_{5}$ and $Er_{6}$ pass the significance test, and the coefficients of the last three are greater than 0, indicating that the “U-shaped” relationship between investment tool and industrial green technology progress in central region is significant. The positive linear relationship between autonomous regulation is established, while command-based regulation has no direct relationship. In terms of the interaction effect, the coefficients of $Er_{3}*Er_{5}$ and $Er_{4}*Er_{6}$ are significantly negative, so there is a “substitution” effect between market-based and autonomous regulatory tool.

Models W1 and W2 show that other variables fail to pass significance test except $Er_{3}$ and $Er_{3}^{2}$, indicating that autonomous and command-based regulations in the western region have no significant impact on industrial green technology progress, and only the “U-shaped” curve effect of the cost-based tool is established. Moreover, the coefficients of the six cross terms are not significant, suggesting that there is no interaction effect between different types of environmental regulation.

In conclusion, in the evolution of industrial green technology, command-based environmental regulation does not work in the three regions. There may be U-shaped effect in central and western regions of market-based environmental regulation, and the linear effect is uncertain in eastern region. The autonomous environmental regulation has a positive promoting effect in the eastern and central regions, but no influence in the western region. The interaction effect between different regulation tools does not exist in the western region, while the substitution effect between market-based and autonomous regulations may exist in the eastern and central regions.

#### 4.3.2. Threshold Regression in the Central and Western Regions

Because of the “quadratic curve” influence of market-based regulation tools in the central and western regions, the threshold effect is carried out. 

Table 11 shows that the central region’s double threshold effect is significantly established, while the western region’s single threshold effect test passed. Therefore, the double threshold model is suitable for the central region, and single threshold model for the western region. Meanwhile the threshold estimates are within the corresponding 95% confidence intervals, so they are authentic. The threshold regression results are shown in Table 12, in which model C3 is for the central region, and model W3 for the western region.

Model C3 shows that there is a threshold effect of market-based environmental regulation on industrial green technology progress due to environmental regulation compliance degree (Ze) in the central region. When Ze≤0.021, the coefficient of $Er_{4}$ is 0.1318. When 0.021<Ze≤0.026, the impact turns to negative and is not significant. When Ze exceeds 0.026, the impact coefficient becomes to 0.1414 and is significant. It is concluded that, with the increasing awareness of environmental regulation compliance in the central region, the effect of investment-based tool on industrial green technology progress has turned from positive to negative and then to positive again. The promotion effects are significant at both sides and the inhibition effect is not significant in the middle part. Autonomous regulation has a positive promoting effect, and the interactive “substitution” effect of autonomous and market-based regulations is significant. It is consistent with the conclusion of the previous model M2. 

Model W3 shows that the single threshold effect of the cost-based tool on industrial green technology progress is characterized by first restrain then accelerate in the western region. When Ze≤0.020, the coefficient of $Er_{3}$ is −0.0032 and insignificant. When Ze>0.020, the impact turns to significantly positive. The influence of other regulation tools and their interaction terms are not significant, which are consistent with the conclusions of the previous model W1.

In summary, the analysis at national and regional levels supports different types of environmental regulation have different effects on industrial green technology progress. The non-linear effects of market-based regulation are significant, and there is a threshold effect of environmental regulation compliance degree. Moreover, there may be a weak substitution interaction between different types of regulations. Hypotheses 1, 2, and 3 are verified.

## 5. Conclusions and Policy Implications

### 5.1. Conclusions

Green technological progress is the focus of environmental regulation. Based on China’s 2000–2018 provincial panel data, this paper uses the EBM-GML model to measure industrial green technology progress and uses a non-linear panel model and threshold panel model to examine the impact of different regulatory tools on industrial green technology progress, considering their interaction and threshold effects. At the same time, the robustness test is carried out from the regulation tool aspect and the regional aspect. The results show that the conclusions are highly stable. The main conclusions are as follows.

First, the impact of environmental regulation on industrial green technology progress varies with different types of regulation. The effect of command-based environmental regulation is not significant, which is the case for the analysis at the national level and the regional level. Except for the western region, autonomous environmental regulation positively promotes the progress of industrial green technology. The effect of market-based environmental regulation is diversified, and the quadratic curve effect is significant at the national level. However, the curves of the cost-based and investment-based tools are different: the former shows an inverted U-shaped, while the latter shows a U-shaped trend. There may be U-shaped evolution in the central and western regions, and the linear relationship in the eastern region is uncertain.

Second, the interaction regression shows that there may be weak substitution effects among different types of environmental regulation. Nationally, there is a negative interaction between market-based and autonomous, and between command-based and autonomous regulations. Regionally, the substitution effect of market-based and autonomous regulations may exist in the eastern and central regions, but not in the western region. It shows that there is no positive synergistic effect between different types of environmental regulation at present, but a weak substitution effect.

Third, the regression of threshold effect shows that the single threshold effect of national market-based environmental regulation is significant. With the improvement of environmental regulations, the impact effect of cost-based tools changes from promotion to inhibition, while that of investment tools changes from inhibition to promotion, which are consistent with the inverted U-sharped and U-sharped quadratic effect. Regionally, there is a double threshold effect of “promotion at both sides and inhibition in the middle” in investment-based tools in central region, and a single threshold effect of “inhibition promotion” exists in the cost-based tool in the western region. In the eastern region, there is no threshold effect.

### 5.2. Enlightenment

Whether environmental regulation can promote industrial green technology progress depends on the implementation and execution of regulatory tools. Based on the aforementioned empirical conclusions, two policy implications are put forward:

(1) Optimize the design of environmental regulation tools. Clarify the supervision responsibilities of relevant environmental laws and regulations, strengthen the targeted supervision of local government environmental policy implementation, and ensure that command-based environmental regulation can truly force enterprises to carry out green technology activities. Timely and proactively increase the intensity of environmental regulation, especially the autonomous and market-based regulation tools, and public supervision and market incentives should be combined to promote enterprises to consciously increase the R&D investment in clean technology, so as to further improve the level of industrial green technology. Evaluate the implementation effect of specific regulation tools, and dynamically adjust the environmental regulation system combined with its policy effectiveness. For example, China’s pollution discharge fee has changed to environmental protection taxes since 2018. Regional economic, industrial and technological innovation differences should be considered comprehensively, to formulate environmental regulation policy system in line with local high-quality development according to local conditions.
(2) Strengthen the coordination of various environmental regulations and mine their complementary effects, such as raising the public’s awareness of environmental protection, regulating the public’s participation in ecological construction from the perspective of legislation, ensuring environmental evaluation information is released timely from the institutional perspective, ensuring the effectiveness of the public in the process of supervision and implementation, and reverse the current negative substitution effect between command-based and autonomous regulation tools into a positive synergistic effect. At the same time, increased public awareness of environmental protection can be coordinated with market-based environmental regulations, thus encouraging enterprises to engage in green technology research and development. In addition, when top managers become more aware of environmental protection, they will give more consideration to environmental benefits and improve the level of green technology while considering economic benefits in the decision-making process of enterprises.

The situation in many developing countries is similar to that in China. The government’s environmental management is rather complicated, and the above conclusions and suggestions can provide references for more developing countries. Therefore, this paper can provide a useful analytical framework for studying the relationship between environmental regulations and technological progress in a country, especially in developing countries.

However, there are still certain limitations in this paper. The impact mechanism of environmental regulation on green industrial technological progress is only qualitatively described, and an in-depth theoretical model analysis has not been carried out. At the same time, based on the analysis of China’s provincial data, it is difficult to reveal the effect of environmental regulation on green technology progress in different industries. In the future, with the improvement and sharing of enterprises micro-data, a more specific analysis can be carried out from the perspective of enterprises. This is also the direction of future research.

## Figures and Tables

**Table 1:** Input–output indicator system.

First-Level Indicator	Second-Level Indicator	Measurement and Notation
Inputs	Capital	Industrial fixed capital stock $\left(X_{1it}\right)$
	Labor	Number of industrial employees $\left(X_{2it}\right)$
	Energy	Industrial terminal energy consumption $\left(X_{3it}\right)$
Outputs	Output value scale	Gross industrial output $\left(Y_{Git}\right)$
	Pollution emissions	Industrial wastewater emissions $\left(Y_{B1it}\right)$
		Industrial SO_2_ emissions $\left(Y_{B2it}\right)$
		Industrial solid waste emissions $\left(Y_{B3it}\right)$

**Table 2:** Three types of environmental regulation tools variable table.

Type	Indicator	Calculation and Variable Notation
Command	Number of laws and regulations issued by local governments	Number of laws issued by local governments + Number of regulations issued by local governments $\left(Er_{1it}\right)$
	Number of environmental administrative punishment cases per capital	Number of provincial environmental administrative penalty cases/Provincial total population $\left(Er_{2it}\right)$
Market	Per capita pollution charges	The amount of provincial pollution fees paid into the treasury/ Provincial total populational $\left(Er_{3it}\right)$
	Intensity of pollution control investment completion	The amount of investment completed in provincial industrial pollution control/ Provincial industrial added value $\left(Er_{4it}\right)$
Autonomous	Number of petitions per capita	Number of provincial petitions (telephone, WeChat, etc.) ^1^/ Provincial total population $\left(Er_{5it}\right)$
	Number of NPC and CPPCC Proposals	Number of provincial National People’s Congress proposals + Number of provincial CPPCC proposals $\left(Er_{6it}\right)$

Since 2011, the number of complaints handled through the telephone and network has been included in the number of petitions per capita; for the period 2016–2018, ^1^ the number of WeChat transactions was also included.

**Table 3:** Descriptive statistics.

Variables	Eastern			Central			Western			China		
	Mean	SD	CV	Mean	SD	CV	Mean	SD	CV	Mean	SD	CV
Kc (%)	1.89	1.40	0.74	1.02	0.37	0.37	0.88	0.57	0.65	1.29	1.04	0.81
Tr (%)	32.39	21.37	0.66	5.86	2.57	0.44	6.56	4.70	0.72	15.84	18.32	1.16
FDI (%)	4.45	2.48	0.56	2.07	1.03	0.50	1.07	0.86	0.80	2.58	2.23	0.87
Zl	2.60	1.83	0.70	1.06	0.87	0.83	0.62	0.59	0.95	1.46	1.52	1.04
S (%)	46.33	11.33	0.24	38.28	5.35	0.14	40.16	4.23	0.11	41.92	8.54	0.20
P	1.74	0.60	0.35	1.72	0.51	0.30	1.63	0.53	0.32	1.69	0.55	0.33
Co (%)	47.68	15.90	0.33	66.32	11.13	0.17	58.73	11.90	0.20	56.70	15.27	0.27
*Er*1 (order)	2.47	2.89	1.17	3.11	4.72	1.52	2.10	4.72	2.24	2.51	4.15	1.66
*Er*2 (case/10,000 person)	1.24	1.52	1.22	0.60	1.34	2.24	0.42	0.29	0.69	0.77	1.22	1.58
*Er*3 (RMB yuan/person)	13.46	7.87	0.58	11.59	12.75	1.10	11.55	10.04	0.87	12.26	10.17	0.83
*Er*4 (%)	0.35	0.24	0.69	0.39	0.29	0.75	0.59	0.47	0.79	0.45	0.37	0.82
*Er*5 (petition/person)	9.83	7.68	0.78	3.80	2.45	0.64	5.90	6.59	1.12	6.78	6.72	0.99
*Er*6 (proposal)	537.2	426.9	0.79	463.9	313.9	0.68	361.5	254.9	0.71	453.2	349.6	0.80
Ze (ton/person)	0.02	0.01	0.30	0.02	0.00	0.22	0.02	0.01	0.31	0.02	0.01	0.30
Gy	1.04	0.10	0.10	1.11	0.24	0.22	1.08	0.20	0.19	1.07	0.18	0.17

SD, standard deviation; CV, coefficient of variation.

**Table 4:** Linear and quadratic regressions of command environmental regulations: Nationwide.

Variables	T11 FE	T12 FE	T21 FE	T22 FE
$Er_{1}$	0.0002 (0.12)	0.0019 (0.79)		
$Er_{1}^{2}$		−0.0001 (−0.84)		
$Er_{2}$			0.0056 (1.35)	0.0035 (0.24)
$Er_{2}^{2}$				−0.0006 (−0.42)
CX R-squared LR test Hausman test Obs	Yes 0.6684 492.77 *** 23.66 *** 570	Yes 0.6693 493.33 *** 19.70 *** 570	Yes 0.6700 491.45 *** 24.74 *** 570	Yes 0.6703 491.01 *** 26.03 *** 570

*T*-values are reported in parentheses; *** indicates statistical significance at 1%; FE indicates fixed-effect models.

**Table 5:** Linear and quadratic regressions of market environmental regulations: Nationwide.

Variables	T31 RE	T32 RE	T41 FE	T42 FE
$Er_{3}$	0.0016 ** (2.03)	0.0043 ** (2.52)		
$Er_{3}^{2}$		−0.0001 *** (−2.80)		
$Er_{4}$			−0.0267 (−1.56)	−0.0987 *** (−2.62)
$Er_{4}^{2}$				0.0437 *** (2.59)
CX R-squared LR test Hausman test Obs	Yes 0.6629 497.80 *** 10.71 570	Yes 0.6673 500.41 *** 13.05 570	Yes 0.6691 485.65 *** 20.69 *** 570	Yes 0.6774 474.838 *** 20.11 *** 570

*T*-values are reported in parentheses; *** and ** indicate statistical significance at 1% and 5%, respectively; FE and RE indicate fixed-effect and random effect models, respectively.

**Table 6:** Linear and quadratic regressions of autonomous environmental regulations: Nationwide.

Variables	T51 FE	T52 FE	T61 FE	T62 FE
$Er_{5}$	0.0018 * (1.69)	0.0026 (0.99)		
$Er_{5}^{2}$		−0.0001 (−0.33)		
$Er_{6}$			0.0001 * (1.74)	0.0001 * (1.79)
$Er_{6}^{2}$				−0.0001 (−0.60)
CX R-squared LR test Hausman test Obs	Yes 0.6719 487.75 *** 22.18 *** 570	Yes 0.6721 486.03 *** 23.19 *** 570	Yes 0.6685 494.55 *** 47.68 *** 570	Yes 0.6686 494.86 *** 30.21 *** 570

*T*-values are reported in parentheses; *** and * indicate statistical significance at 1% and 10%, respectively; FE indicates fixed-effect models.

**Table 7:** Regressions of interaction effect of different environmental regulations: Nationwide.

Variables	T1 FE	Variables	T2 RE
$Er_{1}$	0.0016 (0.98)	$Er_{2}$	0.0042 (0.45)
$Er_{3}$	0.0076 *** (3.49)	$Er_{4}$	−0.1064 ** (−2.07)
$Er_{3}^{2}$	−0.0001 *** (−2.63)	$Er_{4}^{2}$	0.0425 ** (2.23)
$Er_{5}$	0.0058 *** (3.08)	$Er_{6}$	0.0001 * (1.81)
$Er_{1}*Er_{3}$	−0.0001 (−0.13)	$Er_{2}*Er_{4}$	0.0242 (1.37)
$Er_{1}*Er_{5}$	−0.0002 (−0.61)	$Er_{2}*Er_{6}$	−0.0001 *** (−2.80)
$Er_{3}*Er_{5}$	−0.0003 ** (−2.57)	$Er_{4}*Er_{6}$	−0.0001 (−0.41)
Kc	−0.0083 (−0.36)	Kc	0.0022 (0.12)
Tr	−0.0021 ** (−2.40)	Tr	−0.0028 *** (−3.56)
FDI	0.0239 *** (5.59)	FDI	0.0229 *** (5.51)
Zl	0.0763 *** (8.01)	Zl	0.0719 *** (8.48)
S	−0.0079 *** (−5.82)	S	−0.0073 *** (−5.45)
P	0.0292 ** (2.29)	P	0.0529 *** (4.93)
Co	−0.0028 *** (−3.21)	Co	−0.0016 ** (−1.99)
R-squared	0.6891	R-squared	0.6810
LR test	479.67 ***	LR test	465.65 ***
Hausman test	33.40 ***	Hausman test	3.02
Obs	570	Obs	570

All concepts are not listed; *t*-values are reported in parentheses; ***, ** and * indicate statistical significant at 1%, 5% and 10%, respectively; FE and RE indicate fixed-effect and random effect model, respectively.

**Table 8:** Threshold effect test: Nationwide.

Threshold Variable	Market-ER	Number of Thresholds	F-Statistic	*p*-Value	Threshold Estimators	95% Confidence Interval	
Ze	$Er_{3}$	Single	17.647 **	0.022	0.034	0.019	0.035
		Double	8.694	0.118			
		Triple	7.426	0.124			
	$Er_{4}$	Single	12.477 **	0.032	0.014	0.013	0.020
		Double	4.680	0.170			
		Triple	3.943	0.176			

Results of bootstrap 800 times; ** indicates statistical significant at 5%.

**Table 9:** Threshold regressions of market environmental regulations: Nationwide.

Variables	T3	Variables	T4
$Er_{1}$	0.0011 (0.65)	$Er_{2}$	0.0027 (0.30)
$Er_{3}(Ze\leq 0.034)$	0.0019 * (1.91)	$Er_{4}(Ze\leq 0.014)$	−0.1494 *** (−2.62)
$Er_{3}(Ze>0.034)$	−0.0136 *** (−3.30)	$Er_{4}(Ze>0.014)$	0.0282 (1.06)
$Er_{5}$	0.0041 ** (2.29)	$Er_{6}$	0.0001 * (1.94)
$Er_{1}*Er_{3}$	−0.0000 (−0.13)	$Er_{2}*Er_{4}$	0.0161 (0.94)
$Er_{1}*Er_{5}$	−0.0001 (−0.46)	$Er_{2}*Er_{6}$	−0.0001 ** (−2.09)
$Er_{3}*Er_{5}$	−0.0002 * (−1.66)	$Er_{4}*Er_{6}$	−0.0001 (−1.15)
Kc	−0.0152 (−0.67)	Kc	−0.0009 (−0.04)
Tr	−0.0020 ** (−2.29)	Tr	−0.0021 ** (−2.41)
FDI	0.0260 *** (6.16)	FDI	0.0264 *** (6.31)
Zl	0.0838 *** (8.99)	Zl	0.0806 *** (8.67)
S	−0.0077 *** (−5.73)	S	−0.0074 *** (−5.49)
P	0.0425 *** (3.64)	P	0.0541 *** (4.59)
Co	−0.0029 *** (−3.27)	Co	−0.0030 *** (−3.39)
R-squared	0.7188	R-squared	0.7016
Obs	570	Obs	570

All concepts are not listed; *t*-values are reported in parentheses; ***, ** and * indicate statistical significant at 1%, 5% and 10%, respectively.

**Table 10:** Regression of interaction effect of different environmental regulations: Three regions.

Variables	E1	C1	W1	Variables	E2	C2	W2
	FE	FE	FE		FE	FE	RE
$Er_{1}$	−0.0020 (−0.49)	−0.0019 (−0.41)	−0.0001 (−0.06)	$Er_{2}$	−0.0027 (−0.34)	−0.0170 (−0.36)	0.0794 (1.13)
$Er_{3}$	0.0063 *** (3.45)	0.0069 (1.29)	−0.0123 *** (−3.29)	$Er_{4}$	0.0196 (−0.53)	−0.3930 ** (−2.36)	0.0741 (1.50)
$Er_{3}^{2}$			0.0004 *** (5.57)	$Er_{4}^{2}$		0.1983 ** (2.29)	
$Er_{5}$	0.0038 ** (2.33)	0.0160 * (1.75)	−0.0004 (−0.12)	$Er_{6}$	0.0001 *** (2.86)	0.0001 * (1.73)	−0.0001 (−0.51)
$Er_{1}*Er_{3}$	0.0001 (0.23)	−0.0018 (−1.53)	0.0003 (0.78)	$Er_{2}*Er_{4}$	−0.0213 (−1.37)	0.0308 (0.96)	−0.0843 (−1.11)
$Er_{1}*Er_{5}$	−0.0001 (−0.59)	0.0010 (1.24)	0.0007 (1.39)	$Er_{2}*Er_{6}$	0.0001 (1.40)	0.0001 (0.15)	0.0001 (0.21)
$Er_{3}*Er_{5}$	−0.0003 *** (−3.24)	−0.0005 *** (−3.72)	−0.0002 (−0.88)	$Er_{4}*Er_{6}$	0.0001 (1.00)	−0.0001 * (−1.77)	−0.0001 (−0.49)
CX	Yes	Yes	Yes	CX	Yes	Yes	Yes
R-squared	0.6629	0.8249	0.7578	R-squared	0.6488	0.8424	0.7032
LR test	96.60 ***	60.18 ***	53.63 ***	LR test	92.34 ***	63.97 ***	75.42 ***
Hausman test	52.32 ***	1358.21 ***	32.61 ***	Hausman test	46.87 ***	76.80 ***	3.20
Obs	209	152	209	Obs	209	152	209

All concepts and the coefficients of control variables are omitted; *t*-values are reported in parentheses; ***, **, and * indicate statistical significance at 1%, 5% and 10%, respectively; FE and RE indicate fixed-effect and random effect models, respectively.

**Table 11:** Threshold effect test: the central and western regions.

Region	Threshold Variable	Market-ER	Number of Thresholds	F-Statistic	*p*-Value	Threshold Estimates	95% Confidence Interval	
	Ze	$Er_{3}$	Single	46.431 ***	0.000	0.020	0.019	0.021
Western			Double	5.064	0.110			
			Triple	2.655	0.202			
		$Er_{4}$	Single	9.799	0.120	0.021	0.020	0.023
Central			Double	18.418 ***	0.002	0.026	0.015	0.028
			Triple	9.607	0.234			

Results of bootstrap 800 times; *** indicates statistical significant at 1%.

**Table 12:** Threshold regressions of market environmental regulations: central and western regions.

Variables	C3	Variables	W3
$Er_{2}$	−0.0471 (−1.21)	$Er_{1}$	0.0013 (0.66)
$Er_{4}(Ze\leq 0.021)$	0.1318 * (1.80)	$Er_{3}(Ze\leq 0.020)$	−0.0032 (−1.34)
$Er_{4}(0.021<Ze\leq 0.026)$	−0.0451 (−0.80)	$Er_{3}(Ze>0.020)$	0.0057 *** (2.91)
$Er_{4}(Ze>0.026)$	0.1414 ** (2.33)		
$Er_{6}$	0.0002 *** (2.75)	$Er_{5}$	−0.0064 (−0.84)
$Er_{2}*Er_{4}$	0.0274 (1.11)	$Er_{1}*Er_{3}$	−0.0001 (−0.15)
$Er_{2}*Er_{6}$	0.0001 (1.15)	$Er_{1}*Er_{5}$	0.0010 (1.10)
$Er_{4}*Er_{6}$	−0.0004 *** (−2.64)	$Er_{3}*Er_{5}$	0.0003 (1.34)
CX	Yes	CX	Yes
R-squared	0.8813	R-squared	0.7730
Obs	152	Obs	209

All concepts and the coefficients of control variables are omitted; t-values are reported in parentheses; ***, **, and * indicate statistical significance at 1%, 5% and 10%, respectively;

## Data Availability

The data presented in this study are available on request from China Energy Statistical Yearbook, China Environment Yearbook, China Industrial Economic Statistical Yearbook, China Population and Labor Statistics Yearbook, China Statistical Yearbook, China Statistical Yearbook on Science and Technology and Provinces’ Statistical Yearbooks from 2001 to 2019.
